# Factors predicting satisfaction in outpatient substance abuse treatment: a prospective follow-up study

**DOI:** 10.1186/s13011-020-00275-5

**Published:** 2020-05-24

**Authors:** Katja Kuusisto, Tomi Lintonen

**Affiliations:** 1 Faculty of Social Sciences, Tampere University, Linna Building, room 6093, FI-33014, Tampere, Finland; 2 The Finnish Foundation for Alcohol Studies, c/o THL, PL 30, 00271 Helsinki, Finland

**Keywords:** Substance use, Substance abuse treatment, Satisfaction, Expectations, Client-centred evaluation, Service user, Outpatient, Effectiveness, Outcome

## Abstract

**Background:**

While treatment satisfaction has been associated with better outcomes in substance abuse treatment, there is an obvious need for a more profound understanding of what predicts client’s satisfaction with treatment. This study elucidates factors relevant to treatment outcome measured at follow-up in terms of satisfaction with the treatment received.

**Methods:**

The research was implemented as a multisite study in outpatient clinics (*N* = 7) in southern and western Finland. Data consists of therapists (*N* = 33) and their clients (*N* = 327). Each consenting client beginning a treatment period was accepted as a research subject and all therapists at the clinics in question participated. The study was conducted as part of the clinic’s normal activity. Clients were allocated to therapists according to a randomization list drawn up in advance. Apart from the randomisation and the completion of questionnaires, it did not interfere with the progress of treatment. Follow-up lasted 6 months. Multiple Classification Analysis (MCA) was used through combinations of variables organized by content, e.g. client demographics, previous substance use, therapist’s characteristics and client’s expectations. The analyses were based in part on conventional statistical testing (*t* -test, *χ*^2^-test, ANOVA).

**Results:**

Among 37 independent variables few were statistically significant in the final model. The results suggest that high treatment expectations at baseline are a strong predictor of satisfaction at follow-up. Also, previous substance use predicted treatment satisfaction; people using multiple substances were less satisfied than those taking only one substance. Stronger predictors reduced the statistical significance of those independent variables that were statistically significant in the first analyses. Therefore, therapist’s role in recovery and readiness to change should be also seen as antecedents to treatment satisfaction.

**Conclusions:**

It seems that treatment expectations are fulfilled among those participating in follow-up. Yet many are lost during treatment and by follow-up. Service users have experiential knowledge that differs from professionals’ and policymakers’ knowledge. It is clinically relevant to understand what factors affect client’s satisfaction. Hence, it is possible to identify the population whose treatment should receive the most attention, how the client’s experience, their commitment to treatment, and treatment effectiveness could be improved.

## Background

Taking account of the client’s perspective is important when researching the treatment of substance use [1, 2]. Some work has been accomplished in this area by putting the focus on service users themselves (see e.g. [3, 4]) along with other stakeholders [5] in defining relevant outcome measures. The main idea is that service users have experiential knowledge that differs from professionals’ and policymakers’ knowledge, and this may be vital in posing the right questions in research and in practice. While substance-taking behaviour has been the primary outcome measure for alcohol treatment trials, and will continue to be so, it does not exclude other outcome measures like satisfaction with the treatment received.

In health research, the importance of measuring satisfaction with treatment is well documented [6], but this is not the case in substance abuse treatment [7]. Treatment effectiveness has seldom been measured in terms of client satisfaction, although its importance is on the increase in treatment studies, both medical and psychological [8].

In earlier research, treatment satisfaction has been associated with positive therapy outcomes measured with conventional measures like retention or substance-taking behaviour (see e.g. [7, 9–14]), although opposite claims have also been made (see e.g. [15, 16]). Although many of these studies have obvious strengths, methodological deficiencies (e.g. cross-sectional data; small group of participants; varying client groups) impede conclusions in this area (see [17]). These somewhat contradictory findings rely on the quantity of research published and firmly corroborate the positive connection between satisfaction and other outcome measures. According to recent research, it seems plausible that a positive connection exists [7, 18], and even with more complicated client groups [19]. Outcome indicators seem to be interconnected; client’s expectations on entering treatment and the whole process of treatment contribute to satisfaction with the treatment received and eventually treatment outcome [11, 20, 21].

Satisfaction is an important indicator of quality of services [22–24] and highlights the client’s perspective. The scientific significance of this study with a prospective research design is in providing information on treatment satisfaction as an outcome measure and its predictors selected on theoretical grounds. While accepting the notion about the interconnectedness of these outcome indicators, there is an obvious need for a more profound understanding of what predicts client’s satisfaction with treatment. Adamson and colleagues [9] in their systematic review found antecedents to treatment outcome such as baseline alcohol consumption, dependence severity, gender, treatment history, alcohol-related self-efficacy, motivation, socioeconomic status/income and treatment goal. When studying client characteristics, readiness for change (see e.g. [25]) and anger (see e.g. [26, 27]) have been found to be significant factors as regards treatment outcome. Similarly, therapist characteristics have been found to influence client outcome (see e.g. [28, 29]). While some research has concluded that the influence of these so-called common factors is far more important than therapy methods (see e.g. [30]), other research has repeatedly stressed the need to study the common factors present in the therapy situation but not specific to a specific therapeutic method (see e.g. [31]).

When treatment satisfaction is seen as an antecedent of better substance abuse treatment outcomes in terms, for instance, of retention in treatment or substance-abusing behaviour, it is clinically relevant to understand what factors affect the client’s satisfaction (see [17, 18]). Hence it is possible to identify the population whose treatment should receive the most attention, how the client’s experience could be improved and consequently also, their commitment to treatment and treatment effectiveness.

Even in the absence of an unambiguous definition of satisfaction [24], various measures have been applied to measure it in substance abuse treatment. Although there are some structured measures available such as the Client Satisfaction Questionnaire (CSQ-8) (see e.g. [32, 33]), the Treatment Perception Questionnaire (TPQ) [34], and the Verona Service Satisfaction Scale (SSS-30) [23, 35], they are usually modified and tailored to suit separate purposes (see e.g. [36]). Single-item measurements (see e.g. [14, 20, 37–39]), subscales of comprehensive measures (see e.g. [22]), questionnaires with composite scores (see e.g. [11, 19]) and even both simultaneously [7, 14, 40] have been used, but often treated as single satisfaction scores in the analyses [41]. It has recently been argued that a single general question regarding overall satisfaction is adequate and predicts satisfaction equally well as a composite score or other items used so far. The clinical utility and convenience of a short question is undeniably a major asset [7] and the notion is well supported elsewhere [40].

In this study we take a closer look at treatment satisfaction measured with a one-item self-report measure 6 months after treatment: *How satisfied you are with the help and support received from the therapist in the outpatient clinic?* The responses used a five-point scale (1 = extremely dissatisfied,…, 5 = extremely satisfied). Analytically, the starting point for the empirical analysis is exploratory, using multivariate analysis. The research question is as follows: *What factors predict client’s satisfaction at follow-up with treatment received?*

## Method

### Design and implementation

The research was implemented as a multi-centre study with the participation of 7 outpatient clinics in southern and western Finland. These clinics provide integrated care for substance use problems. In addition to psychosocial services, medication may be one part of the treatment. Rigorous principles were adhered to in the design and implementation of this prospective follow-up study [20]. Thus, a naturalistic research approach was applied, meaning that the research was conducted as part of the clinic’s normal activity. Apart from the randomization of clients to therapists and the completion of questionnaires, it did not interfere with the progress of treatment. A further aim was to use a minimal number of burdensome tools. This was an attempt to minimize the effect of the research on treatment outcomes (see [42]). Clients participated in the study on a voluntary basis and were informed of their right to withdraw from it at any time. Withdrawing from the study did not adversely affect the care they received. Thus, the clients (*N* = 327, 111 women (33.9%), 216 men (66.1%)) were non-selected; each consenting client beginning a new treatment period due to a substance use problem was accepted as a research subject. All clients who had completed the baseline questionnaire were invited by letter to a follow-up appointment at the clinic 6 months after the start of treatment. No information was gathered on clients who declined to participate.

All therapists (*N* = 33) at the clinics in question participated. Information on the therapists was also gathered and aligned with client data. Clients were allocated to therapists according to a randomization list drawn up in advance to standardize background information. Client’s treatment outcome was monitored 6 months after commencement of treatment; it was the only specifically determined meeting in the procedure. The times for therapy sessions were determined by the client’s needs and the practices of the unit in question.

**Fig. 1:**
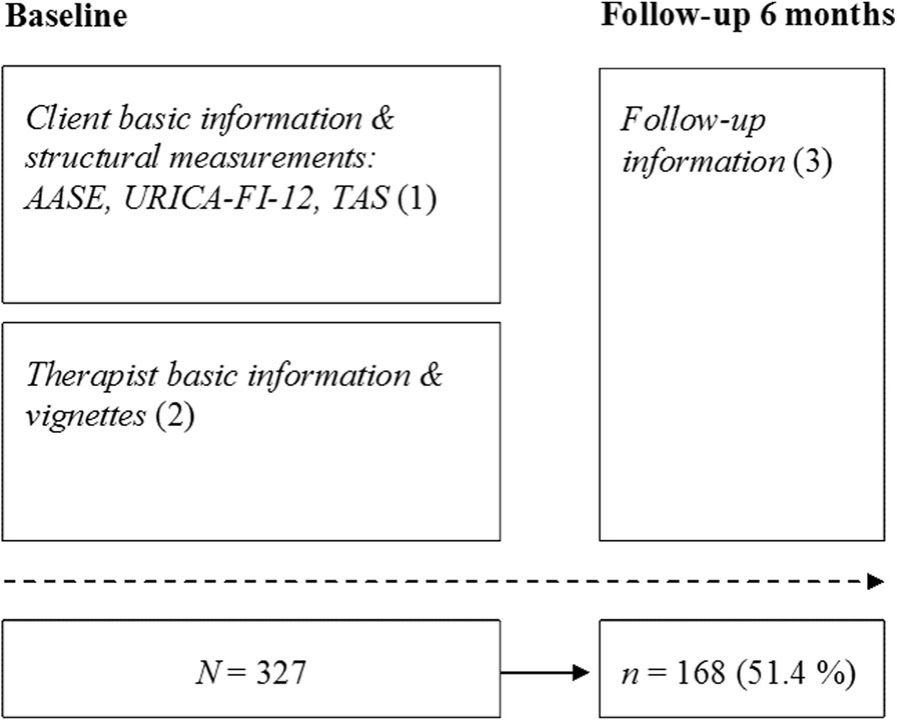
Research procedure and number of subjects at different stages. Figure presents the progress of the study, the research materials used and numbers of clients at two measurement points (baseline and at 6-month follow-up). Clients completed a questionnaire and structural measurements (marked with: 1). At the beginning of the research the background information of the therapist and therapeutic orientation were also elicited (2). All clients who completed the form for background information were invited by letter 6 months after the beginning treatment for a follow-up visit at the unit. The treatment follow-up questionnaire was completed (3). The measurements are described in greater detail in the maintext. Number of clients at baseline (*N* = 327) and at 6-month follow-up (*n* = 168) are presented

Figure 1 presents the progress of the study, the research materials used and numbers of clients in the various phases. Client participation began with the first visit to the unit’s reception. Having received a brochure describing the research, a client gave consent to being a research subject. The client was apprised of the research ethics. In this study we adhered to the guidelines of the Finnish codes of research ethics and governance [43, 44] and with the codes of research integrity in Europe [45] and the guidelines of the Finnish professional systems. This means among other things that participation was voluntary and anonymity of participants was protected.

### Questionnaire and measurement instruments

Before commencing actual therapy clients completed a questionnaire (Fig. 1; 1), which combined questions eliciting e.g. demographic factors, information on substance use and attitudes to treatment. The questionnaire incorporated combined questions reportedly used in other Finnish studies [46, 47] and some structural measurements. The measurements used have been described in greater detail elsewhere [20, 21, 39, 48].

Data on independent variables was collected as part of the baseline questionnaire package, namely 6 months before data on treatment satisfaction was collected. Client self-efficacy was measured by the 12-item version of Alcohol Abstinence Self-Efficacy Scale (AASE) [49]. The clients were given descriptions of various situations and moods, and were asked to use a five-point scale to describe how likely they were to have refused the use of substances during the past week (1 = extremely unsure,…, 5 = extremely sure). The alpha (α) reliabilities for the four scales and composite variable combining the scales were on the same level as in the original study (in parentheses): negative affect 0.80 (0.82), social/positive 0.83 (0.82), physical and other concerns 0.80 (0.83), craving and urges 0.81 (0.81) and composite variable 0.91 (0.91). The University of Rhode Island Change Assessment Scale (URICA) [50] was used to measure client’s readiness to change. The Finnish 12-item version of URICA-FI-12 was used [46, 47]. The clients responded to the URICA on a five-step numerical scale (1 = totally disagree,…, 5 = totally agree). The items were divided into four scales. The α reliabilities of the scales were on the same level as in the original study (in parentheses): pre-contemplation 0.71 (0.69), contemplation 0.76 (0.75), action 0.72 (0.82), maintenance 0.79 (0.80). The client’s anger was elicited using the 10-item Trait Anger Scale (TAS), which is a part of the State-Trait Anger Expression Inventory-2 (STAXI-2) [26]. The clients responded on the form using a four-step numerical scale (1 = hardly ever,…, 4 = almost always). The composite score for the scale was used in the study and the α reliability was comparable to the measure of the STAXI-2 manual,^1^ being 0.85 in the study at hand.

At the beginning of the research the background information of the therapist and therapeutic orientation were also elicited (Fig. 1; 2). Therapist’s interpersonal functioning was investigated at the same time using the method of Valle [51], where the rating criteria originated in Carkhuff and Berenson [52]. Inter-rater reliability measured by Cronbach’s α was good and inter-vignette reliability (in parentheses) even higher: empathy 0.81 (0.87), genuineness 0.71 (0.90), respect for client 0.76 (0.90), concreteness 0.81 (0.89).

The research included a maximum of five sessions per client. A treatment period might continue thereafter but these subsequent sessions were no longer included in the study. All clients who completed the form for background information were invited by letter 6 months after the beginning treatment for a follow-up visit at the unit. The treatment follow-up questionnaire (Fig. 1; 3) elicited among other things satisfaction with help or support received from the therapist. The dependent variable, satisfaction with treatment, was elicited in a questionnaire at 6-month follow-up. The measurement was based on client self-report on a scale 1–5.

### Statistical analyses

The analyses were carried out with Multiple Classification Analysis (MCA) (see e.g. [53, 54]), multi-method procedure which allows using multiple independent variables to explain variance in a dependent variable. It combines the main properties of analysis of variance (ANOVA) and regression analysis. MCA is considered to be a more robust method than regression analysis [55, 56]. Compared to ANOVA, MCA gives direct information not only on group differences but also on where these differences emerge. In the large dataset at hand, using MCA makes it possible to find multiple independent variables with explanatory power regarding the response variable, treatment satisfaction. In MCA it is possible to control for multiple background variables simultaneously and to examine changes in group differences while other variables are controlled for. In this situation, the analysis reveals the effect of background variables on the phenomenon of interest. MCA produces group specific means of the impact of these background variables on this phenomenon. In MCA the comparison is performed with respect to the average of the data as a whole. The statistically significant independent variables (with *p*-value <.05) for treatment satisfaction were sought and their predictive value was estimated.

The analysis advanced through combinations of variables organized according to content; in other words, treatment satisfaction was predicted by thematic, hierarchical blocks [57]. Using of these hierarchical blocks was essential because large numbers of variables cannot be included at the same time. The analyses were done with MCA in hierarchical blocks (variables in parenthesis): *client demographics and other baseline information* (age group; gender; marital status; living with children; education; housing; employment status), *client’s substance use at baseline* (type of substance use; habit of using substance; duration of prolonged abstinence period; abstinent days during past 30 days; contacts with problem users; prior admission to the clinic in question), *therapist’s interpersonal functioning* (directiveness; empathy; concreteness; therapist’s total skills), *client’s expectations* (attitudes toward Alcoholics or Narcotics Anonymous, AA/NA; religiosity; voluntariness of admission; objectives regarding substance use; preferred gender of therapist; importance of medication; expectations regarding treatment outcome; therapist’s role in recovery), *client’s characteristics* (readiness for change, URICA; self-efficacy, AASE; anger, TAS). Variables expressing *client’s substance use at follow-up* (type of substance use; abstinence vs. controlled use; habit of using substance; duration of prolonged abstinence period; abstinent days during past 30 days; contacts with problem users; AA/NA attendance; other substance abuse treatment attendance) were also included in the analysis in order to study their effect on treatment satisfaction. Each hierarchical block was first analysed separately against the response variable using MCA. Statistically significant variables within these analyses were later considered as key independent variables in the MCA model.

Satisfaction with treatment received is attributable to natural causes (on a scale 1–5, *M* = 4.24; *SD* = 0.968) and is more centred around “more satisfied” than “less satisfied”, which is often the case in social sciences in general and also characteristic of measuring treatment satisfaction [38, 58].

Missing data is mostly due to the prospective research design of the study and non-response at 6-month follow-up. Attrition occurs when participants’ treatment retention is discontinued or when they choose not to participate in follow-up. No imputations were made, but attrition was analysed. The attrition analyses were based on conventional statistical testing (*t* -test, *χ*^2^ -test, ANOVA) comparing differences in independent samples.

### Participants’ characteristics

Table 1 presents information on clients’ backgrounds and substance use. Regarding the demographics, it can be stated in general that the clients’ level of education was decidedly low and the level of unemployment correspondingly high. All in all, the demographic information largely corresponded to the picture provided by earlier Finnish research regarding clients in substance abuse treatment in the community [59].

**Table 1:** Participants’ (*N* = 327) background and substance abuse information

	*M*	*SD*	*n*	%
Age (years)	43.1	11.4		
Marital status				
Single			211	64.5
Pair relationship			116	35.5
Education				
Comprehensive school not completed			87	26.6
Comprehensive school			163	49.9
Upper secondary school			77	23.5
Employment status				
Employed			139	42.5
Not employed			188	57.5
Housing				
Owner-occupier			100	30.6
Tenant			192	58.7
Homeless			35	10.7
Substance used ^a b^				
Alcohol			315	97.5
Benzodiazepines			60	18.6
Cannabis			44	13.6
Amphetamine			37	11.5
Opioids			28	8.7
Cocaine			8	2.5
LSD			5	1.5
Others (incl. Substitute alcohol, solvents)			11	3.3
Type of drug use ^a^				
Single-substance use			236	73.3
Poly-substance use			86	26.7
Habit of using substance ^a^				
Daily			125	39.3
Periodically			127	39.9
At weekends			66	20.8
Duration of continuous abstinence period preceding admission (days) ^a^				
0–7			57	17.4
8–30			119	36.4
31–			151	46.2
Abstinent days during past 30 days				
0–7			68	20.8
8–14			54	16.5
15–22			84	25.7
23–			121	37.0
Contacts with problem users ^a^				
Daily or almost daily			47	14.4
Weekly			83	25.4
Monthly			53	16.2
Less frequently			47	14.4
No contacts			97	29.6
Attitudes towards AA/NA				
Positive or very positive			185	56.5
Neutral			115	35.2
Negative or very negative			27	8.3
Prior admission to the clinic				
Yes			148	45.5
No			177	54.5
Voluntary admission				
Yes			241	73.9
No			85	26.1
Client’s objective				
Abstinence			131	40.7
Controlled use			191	59.3

Note. ^a^ For the year prior to treatment; ^b^ Percentages are measured of the total amount of the participants (*N* = 327) in every value of the variable

Alcohol was the primary substance used, and there was a tendency towards the use of only one substance. As supplements to alcohol the most commonly used substances were benzodiazepines, cannabis and amphetamine. It is illustrative of the substance use problems that the consumption of only one fifth of the clients was limited to weekends. Almost half of the clients had had previous contacts with the clinic in question. Non-voluntary admission (26%) consisted of people entering outpatient treatment because of e.g. driving under the influence or requirements imposed by their workplace. On the other hand, the ability to control consumption emerged: approximately one fifth (19%) of clients had been totally abstinent for the last month before commencing treatment. In earlier international treatment research attention has likewise been paid to the fact that consumption is reduced prior to entering treatment [60, 61].

In line with earlier studies (see e.g. [39, 48]), almost half of the therapists had professional higher education. Two thirds of them were social workers (64%), while the rest were registered nurses. However, their job in all cases was therapy work with clients. Most of the therapists had worked in substance abuse treatment for a considerable time, 46% for 5–15 years and 30% for over 15 years. Methodological eclecticism was most common; 61% of therapists used combinations of different methods. Of the single technical approaches used by the therapist, cognitive therapies (12%), solution-focused therapies (12%), motivational interviewing (3%), and psychodynamic orientation (3%) emerged. Almost half of the therapist (46%) had lengthy training in therapy.

### Attrition analysis

Although clients were randomly assigned to the therapists, attrition was analysed by two types of comparison. In order to exclude their effect, differences were examined by controlling for background variables selected on theoretical grounds. These included e.g. age, sex, previous contact with the treatment unit and client’s objectives for future substance use (abstinence/moderate use).

First, baseline variables were compared between clients who entered treatment unit without further entering therapy (*n* = 41) and those who entered therapy provided by the therapist (*n* = 286) differed from each other in terms of their expectations of treatment outcome, *t*(327) = − 2.489, *p* = .013. Those entering therapy had more positive expectations regarding treatment outcome.

Second, the same variables were used to compare clients who participated in the follow-up (*n* = 168) and those who did not (*n* = 159). There were significant differences in the following variables: age, *t*(325) = 3.464, *p* = .001; housing, *χ*^2^ (2, 327) = 15.353, *p* < .001 and using multiple substances, *χ*^2^ (1, 322) = 7.043, *p* = .008.^2^ The clients who participated in the follow-up were about 4 years older than those who did not. They were also more often house owners or occupiers rather than living in a rented apartment, and they were more seldom homeless than the dropouts. Poly-substance use increased the likelihood of not attending follow-up. However, controlling for age largely explained the differences. The difference in housing was explained by the effect of age; younger clients lived in more deficient accommodation and were using multiple substances more often than were the older clients. These variables were considered as possible confounders in the final MCA model. Overall, it appeared that the stability of life context increased with increasing age and less problematic substance consumption supported treatment retention after treatment had begun. The client’s subjective reasons for not attending the follow-up appointment were not empirically assessed.

## Results

### Treatment satisfaction

Treatment satisfaction mean within the sample was *M* = 4.24 on a five-point scale which seems relatively high. It should be taken into account that those who evaluated treatment satisfaction were those who participated in the follow-up (*n* = 168; 51.4%). It is highly likely that they are above average in evaluating treatment satisfaction compared to those who dropped out during treatment.

Some hierarchical blocks (*client demographics and other baseline information, therapist’s interpersonal functioning*) did not predict satisfaction. Statistically significant predictors were found in subsequent hierarchical blocks: *client’s substance use at baseline, client’s expectations, client’s characteristics and client’s substance use at follow-up*. Type of substance use (single- or poly-substance use) measured at baseline (*F*[1, 157] = 9.583, *p* = .002), expectations on treatment outcome: i.e. positive outcome will occur during the following 6 months (*F*[4, 160] = 3.753, *p* = .006) and therapist’s role in reco-very (*F*[4, 160] = 3.390, *p* = .011) predicted treatment satisfaction, as also did readiness for change (*F*[2, 162] = 4.923, *p* = .008). Type of substance use at follow-up was also statistically significant (*F*[1, 129] = 7.135, *p* = .009).

All variables found significant were included in the next model, which led to the deletion of two variables with no statistical significance in the model. Client attribution of recovery to the therapist was first removed from the analysis with non-significant *p*-value (.350). The predictive value of the model^3^ was *R*^*2*^ = .224. The variable “type of substance use at follow-up” with a *p*-value (.105) was next eliminated. The predictive value of the model increased slightly, *R*^*2*^ = .229. Type of substance use was measured similarly at baseline and at follow-up. They showed a mutual correlation despite the temporal difference between them.

Before the attribution variable was removed as a weakest independent variable, “readiness for change” was not yet statistically significant and its *p*-value was close to the attribution variable. Because the variables removed were statistically significant in previous analyses it was considered useful to analyse these variables more closely. Examination of the model was done by adding statistically significant variables one after another to the original *client’s expectations* block and gradually removing non-significant variables. In the course of this procedure, changes in the significance levels of the variables and predictive models were scrutinized. It appeared that readiness for change and attributing success to the therapist were almost uniform in explaining treatment satisfaction, but when both were entered into the model, they were not statistically significant. As a multivariate analysis, MCA seems to have similar properties since the assessment concerns the unique contribution of one variable over the rest; the strength of one explanatory variable may cause another borderline significant variable to become non-significant. This extension of the analysis proved constituent and revealed that how ready the client is to change his/her behaviour and how much the client attributes recovery to the therapist are both extremely relevant and should be included in examining treatment satisfaction. Therefore, the final model also included both these variables. At the final stage, statistically significant variables from the second attrition analysis (age-group; housing) were included in the model to study their potentially confounding effects. The variables included in the final model are presented in Table 2 below.

**Table 2:** Variables predicting satisfaction with the treatment received at 6-month follow-up by Multiple Classification Analysis (MCA)

Variable	*N*	Baseline mean satisfaction score	Unadjusted deviation	Eta	Predicted adjusted mean satisfaction score	Adjusted deviation	Beta	
Type of substance use (*n* = 158)								
Single-substance use	126	4.40	0.131		4.36	0.091		
Poly-substance use	32	3.75	−0.516		3.91	−0.358		
				.272			.189	*
Expectations on treatment outcome: positive outcome during the 6 months (*n* = 158)								
Extremely unsure	11	3.73	−0.539		3.91	−0.361		
Unsure	9	4.33	0.068		4.40	0.133		
Neutral	49	3.88	−0.388		3.91	−0.353		
Sure	55	4.38	0.116		4.37	0.103		
Extremely sure	34	4.79	0.528		4.69	0.424		
				.379			.315	**
Readiness for change (*n* = 158)								
Low	37	4.00	−0.266		4.07	−0.198		
Medium	72	4.19	−0.071		4.22	−0.041		
High	49	4.57	0.306		4.48	0.210		
				.228			.161	
Attributing to therapist (*n* = 158)								
1 (low)	22	4.18	−0.084		4.12	−0.142		
2	37	3.81	−0.455		3.96	−0.310		
3 (medium)	73	4.40	0.131		4.35	0.086		
4	22	4.55	0.280		4.58	0.310		
5 (high)	4	5.00	0.734		4.64	0.374		
				.299			.223	
Age-group (*n* = 158)								
Under 30	19	3.84	−0.424		4.16	−0.106		
31–40	26	4.12	−0.150		4.24	−0.030		
41–50	53	4.32	0.055		4.30	0.031		
51 and over	60	4.42	0.151		4.29	0.019		
				.195			.047	
Housing (*n* = 158)								
Owner-occupier	64	4.32	0.057		4.29	0.027		
Tenant	82	4.18	−0.083		4.16	−0.106		
Homeless	11	4.55	0.280		4.90	0.631		
				.106			.192	*
Predictive value of the model (*R*^2^)							.299	

Note. Significance in adjusted situation * *p* < .05, ** *p* < .01

There were baseline differences in all variables included in the final model (Table 2). Once standardized, the differences remained similar. This speaks for the clear independent effect of these variables on treatment satisfaction. The best predictor of satisfaction with the therapist was outcome expectations at baseline: those participants with higher expectations of a positive outcome during the following 6 months were more satisfied with the treatment received. However, the connection was not perfect. A small population of those who were unsure regarding their expectations were quite satisfied. MCA as a method of analysis is good in capturing nonlinear connections. The results also showed that people using a single substance evaluated help and support received from the therapist more positively than did those using multiple substances. Those who were homeless at baseline evaluated satisfaction with the therapist more highly than others. High readiness for change also predicted treatment satisfaction, although in the final model it was not a statistically significant predictor. Attributing recovery to the therapist was similarly a non-significant predictor in the final model: the more success the client attributed to the therapist at baseline the more satisfied he/she was at follow-up. The predictive value of the final model was *R*^*2*^ = .299 (i.e. 30% of total variance).

Finally, we checked for a possible correlation between satisfaction and other outcome variables available in this study. Satisfaction did not correlate with retention in treatment. Instead, treatment satisfaction and percentage of days abstinent at follow-up had a positive, statistically significant correlation (*r* = .262, *p* = .001); treatment satisfaction grows as abstinent days increase.

## Discussion

This study was concerned with outpatient substance abuse treatment clientele and their satisfaction with treatment at follow-up. Given the nature of prospective follow-up data, we were able to answer to some of the key demands for future research set in earlier studies in determining the causal relationships between determinants and treatment satisfaction (see e.g. [17, 62]). Our findings add to the knowledge of previous studies about the factors related to client satisfaction in the Finnish outpatient substance abuse treatment system. It appeared that only few indicators had explanatory power regarding treatment satisfaction. The participants’ expectations regarding the therapist’s importance for their recovery had an effect on how they felt supported by their therapist at follow-up; satisfaction with the therapist was greatest when the pre-treatment expectations had been high. Alone, in a standardized situation, it explained almost 32% of variation in satisfaction with the treatment received. The results show that treatment expectations are a strong predictor of satisfaction at follow-up (see e.g. [21]). It seems that expectations regarding treatment for those clients who persevere with the treatment are fulfilled and that this becomes apparent in their evaluations of their satisfaction at follow-up.

Past substance use predicted treatment satisfaction. When standardized, it explained almost 19% of variation in treatment satisfaction. People using multiple substances were less satisfied than those using only one substance. Prior substance use has been found to be a significant predictor of treatment outcome in numerous studies [9, 18]. When the substance use problem was more complicated the client was less satisfied at follow-up. In this study, the stability of life context and how problematic the substance abuse had been before entering treatment also affected the attrition rate. In this respect, the predictive role of the complexity of the substance use problem in terms of prior use supports this same phenomenon: the more complex the problem, the greater the discrepancy between the client’s expectations and the treatment received (see e.g. [18]) The findings suggest that those who are most satisfied are the ones with the mildest problems. This relationship may be inverse; perhaps those respondents whose substance use is less problematic are more likely to be satisfied and compliant. It must be noted that a fairly small share of those clients who had been homeless at baseline and managed to participate in 6-month follow-up were extremely satisfied with the treatment they had received. There may have been a substantial improvement in their life situation. Housing explained 19% of variation in treatment satisfaction. Still, many were lost during treatment as the attrition analyses showed. Either way, the complexity of the substance use problem inevitably makes treatment more important. Nevertheless, according to this study, outpatient treatment has not been successful enough with this client group. An effort should be made to study how the treatment and retention of those clients with multiple problems could be improved. Both of these may reflect the important role of client’s expectations in addition to the actual treatment received (see e.g. [63]).

It is noteworthy that a large number of indicators did not seem to explain the variance in treatment satisfaction. We analysed 37 independent variables but only a few of them were statistically significant while others had no predictive power regarding satisfaction. For example, client demographics or therapist’s interpersonal functioning did not explain satisfaction with the help and support received from the therapist. Also, stronger predictors reduced the statistical significance of those independent variables that were statistically significant in the blocks. While testing these non-significant variables in a hierarchical block of *client characteristics*, readiness for change as the sole statistically significant variable predicted 24% of treatment satisfaction. Similarly, of *client’s expectations,* those attributed to the therapist predicted over 29% of the variance in treatment satisfaction. Therefore, the type of substance use at baseline and at follow-up, expectations regarding treatment outcome, therapist’s role in recovery and readiness to change should be all seen as antecedents to treatment satisfaction even though they were not statistically significant in the final model. In this regard, the variables were quite strongly associated with treatment satisfaction and thus the results represent a clinically meaningful relationship when studying the antecedents of satisfaction with treatment received.

It is also interesting that variables related to treatment satisfaction were primarily client-related rather than therapist-related. Our findings did not reveal many “quality of care” type measures (e.g. therapist’s interpersonal functioning) to be associated with satisfaction. Although therapist’s interpersonal characteristics, such as empathy or directiveness, have proven essential factors in terms of positive treatment outcome (e.g. [28, 29]), satisfaction seems more related to the client’s own pre-treatment expectations and views concerning treatment. Pre-treatment confidence in treatment or therapist has been found to predict good treatment outcome. Confidence is constructed as a combined effect of the client’s expectations regarding treatment and regarding themselves. (e.g. [20, 64]).

In addition, it is an advantage that this study was not profiled as a customer enquiry, which treatment units occasionally implement. The scientific impact of this study was in discovering factors that affect treatment satisfaction for those who persevere with their treatment. Systematic evaluation of treatment outcomes is increasingly becoming a customary procedure in therapeutic treatment in order to respond to emerging requirements imposed by funders, but also to inform clinical decision-making during treatment and to improve treatment effectiveness and client participation [22, 65–67].

### Limitations

The principles considered strengths in this study – i.e. prospective follow-up data gathered in naturalistic settings from the non-selected clients in several outpatient units in Finland - are dealt with in the section on methods. The study, however, has certain limitations that need to be borne in mind when evaluating the findings. The measurement of treatment satisfaction was based on the client’s own assessment. An assessment of a single-item on a scale of one to five may, however, be too crude to optimally reflect the complexity of satisfaction, although single-item measurement has been regarded as both empirically and clinically valid [7]. Maybe a visual analog scale with low estimates to the left and high estimates to the right producing a continuous variable could be useful in examining changes in satisfaction over time by differentiating and broadening client’s experiences [62]. By also being client-centred, measuring client satisfaction can be justified in that it serves additionally as an important measure of quality of care [14].

The group participating at follow-up was quite homogenous in terms of satisfaction. The treatment satisfaction mean was relatively high in this study. This may be due to the ceiling effect often observed in treatment satisfaction studies [38], when participants’ scores cluster toward the high end of the measure. This may also explain why therapist-related factors did not explain client satisfaction. Dropping out of treatment can be treated as an indicator of not being satisfied. Clients tend to “vote with their feet” when the therapeutic alliance is not satisfactory and the treatment received does not meet the client’s expectations [21, 39]. These people who drop out do not usually end up giving evaluations of the treatment. In this study, the follow-up rate of the study remained at 51.4%; almost half of the participants were lost to follow-up. Because of the attrition analyses performed, we know that clients with more stable life situations and less problematic substance use most likely continue with their treatment. Presumably the reasons for drop-out may vary from continued substance use to endeavours to recover naturally without treatment. Satisfaction or dissatisfaction with the help and support received from the therapist may be either uniform or varying between these groups.

The follow-up lasted 6 months and a maximum of 5 sessions per client. The duration of the follow-up period can be justified by the dynamics of addiction, in that relapses most typically occur during that period [68]. Nevertheless, additional information on the clients’ subsequent coping would have been useful, although it was not possible to collect it within the project schedule. Treatment satisfaction, like other outcome measures, can access only one part of the entirety of treatment effectiveness. Diversity in outcome measures still pays off in treatment research. Treatment satisfaction should also be complemented with other indicators measuring treatment effectiveness (see e.g. [69]).

Within this study, nested design might have yielded additional information on between-therapist variation or differences between treatment units regarding their clients’ satisfaction. Due to the statistical software used, our ethical commitment not to compare different treatment units and our research goal to basically find predictive variables to treatment satisfaction, this was not studied. Also, it is entirely plausible that qualitative research methods could contribute to research in bringing the client’s experience into focus (see. e.g. [3]).

## Conclusions

Many stakeholders expect treatment to be effective. Do these findings suggest that treatment satisfaction is not an important issue? Since this is a population that we know may not be satisfied, should we not engage in measuring this as researchers, clinicians and politicians?

Nowadays, researching treatment satisfaction is a choice emphasizing client-centredness (see e.g. [2, 60]). While it is important to obtain information about the factors affecting treatment effectiveness in terms of client satisfaction [7], on the other hand, it is important to address those problems caused by the demands of effectiveness, especially within substance abuse treatment. It has to be borne in mind that within the social services, the effectiveness of services does not always coincide with the disappearing of the client’s service needs. The problems are often complex and progress in treatment is slow.

When the demands of effectiveness and evaluation of services are seen as top-down administrative tools, they do not give a full picture of what is carried out at grass-roots level or of how these affect the clients’ lives. Paying attention to the client’s point of view renders the examination of effectiveness ethically sustainable. Systematic evaluation of treatment outcomes is becoming an increasingly customary procedure in therapeutic treatment in order to respond to emerging requirements imposed by funders, but also to inform clinical decision-making during treatment and to enhance treatment effectiveness and client participation [65–67]. The connection between client’s positive rating of the early therapeutic alliance and client’s satisfaction with treatment received has been reported in our earlier studies [39]. Information about clients’ satisfaction with treatment can serve as a simple clinical tool with clear practical implications. It can help to develop treatment procedures and to improve the performance of treatment institutions, thereby contributing to social reintegration.

## Data Availability

Finnish Social Science Data Archive (FSD) handles the long-term preservation and dissemination of the research data. Data is accessible in accordance with the terms and conditions agreed upon by the depositor and the archive, and are available with approval from the corresponding author on reasonable request. Persistent identifiers: therapist data (urn:nbn:fi:fsd:T-FSD2688) and client data (urn:nbn:fi:fsd:T-FSD2687).

## Abbreviations

AA: Alcoholics Anonymous
AASE: The Alcohol Abstinence Self-Efficacy Scale
ANOVA: Analysis of Variance
CSQ-8: The Client Satisfaction Questionnaire-8
MCA: Multiple Classification Analysis
NA: Narcotics Anonymous
STAXI-2: State-Trait Anger Expression Inventory 2
TAS: Trait Anger Scale
TPQ: Treatment Perceptions Questionnaire
URICA: University of Rhode Island Change Assessment Scale
URICA-12-FI: University of Rhode Island Change Assessment Scale, the Finnish 12-item version
α: alpha

## Footnotes

1 The reliability of the scale is only reported according to gender (0.86 for males, 0.84 for females) in Spielberger [26].

2 The tests of significance were carried out before a specifying elaboration. After this, the stability of inter-variable variance and the variation in the different categories were controlled.

3 *R*^2^ can be interpreted as a percentage of how much of the overall variance in satisfaction with treatment is explained by the selected explanatory variables included in the model.
